# Impairment of corneal epithelial wound healing is association with increased neutrophil infiltration and reactive oxygen species activation in tenascin X-deficient mice

**DOI:** 10.1038/s41374-021-00576-8

**Published:** 2021-03-29

**Authors:** Takayoshi Sumioka, Hiroki Iwanishi, Yuka Okada, Masayasu Miyajima, Kana Ichikawa, Peter S. Reinach, Ken-ichi Matsumoto, Shizuya Saika

**Affiliations:** 1 Department of Ophthalmology, Wakayama Medical University School of Medicine, Wakayama, Japan; 2 Department of Ophthalmology, Wenzhou Medical University, Wenzhou, P. R. China; 3 Department of Biosignaling and Radioisotope Experiment, Interdisciplinary Center for Science Research, Organization for Research and Academic Information, Shimane University, Izumo, Enya-cho Japan

**Keywords:** Cell signalling, Signal transduction

## Abstract

The purpose of the study was to uncover the role of tenascin X in modulation of healing in mouse corneas subjected to epithelium debridement. Healing in corneas with an epithelial defect was evaluated at the levels of gene and protein expression. Wound healing-related mediators and inflammatory cell infiltration were detected by histology, immunohistochemistry and real-time RT-PCR. Tenascin X protein was upregulated in the wounded wild-type (WT) corneal epithelium. The lack of tenascin X impaired closure of an epithelial defect and accelerated infiltration of neutrophils into the wound periphery as compared to the response in WT tissue. Expression of wound healing-related proinflammatory and reparative components, i.e., interleukin-6, transforming growth factor β, matrix metalloproteinases, were unaffected by the loss of tenascin X expression. Marked accumulation of malondialdehyde (a lipid peroxidation-derived product) was observed in KO healing epithelia as compared with its WT counterpart. Neutropenia induced by systemic administration of a specific antibody rescued the impairment of epithelial healing in KO corneas, with reduction of malondialdehyde levels in the epithelial cells. Finally, we showed that a chemical scavenging reactive oxygen species reversed the impairment of attenuation of epithelial repair with a reduction of tissue levels of malondialdehyde. In conclusion, loss of tenascin X prolonged corneal epithelial wound healing and increased neutrophilic inflammatory response to debridement in mice. Tenascin X contributes to the control of neutrophil infiltration needed to support the regenerative response to injury and prevent the oxidative stress mediators from rising to cytotoxic levels.

## Abstract (web summary)

Tenascin X-deficient mice exhibit impaired epithelial closure with accelerated infiltration of neutrophils into the tissue and an increased oxidative stress, when compared to wild-type mice. The impairment of epithelial healing in tenascin X-deficient mice was rescued by targeting neutrophil circulation and reducing reactive oxygen species using a chemical scavenger.

## Introduction

The corneal epithelium is the outermost tissue layer of the eye and its intactness is essential for normal vision. This tissue provides numerous supportive functions, which depend on its ability to undergo continuous renewal. They include providing tight junctional barriers to pathogenic infiltration, eliciting osmotic-coupled fluid extrusion into the tears from the underlying stroma, which helps maintain corneal thinness and transparency. Subsequent to disruption of epithelial integrity by wounding it is essential that reepithelialization rapidly resurface a defect. This is necessary to reduce the likelihood of pathogenic infiltration into the underlying stroma, which can result in corneal swelling as well as damage to the basement membrane and innervation. Other outcomes can include inflammation and scarification, which can result in opacification and loss of tissue transparency. To counter the consequence of these sight compromising events, the corneal epithelial layer must be rapidly reepithelialized to restore corneal transparency and normal vision. Corneal epithelial repair occurs in two different phases, initially involving increases cell spreading and migration toward the wound center followed by contributions from increases in cell proliferation at the wound periphery [1–3]. In order to lessen the likelihood of chronic corneal pathology resulting from injury to the epithelial layer, numerous studies are focused on identifying novel targets whose modulation can hasten the epithelial wound healing response [4–7].

Various growth factors/cytokines are upregulated during epithelial healing upon injury and orchestrate the timing and increases in cell migration and proliferation driving this response [1–3]. Transforming growth factor β (TGFβ) upregulation and activation of its cognate receptor linked to both the p38 MAPK and the Smad3 signaling pathway axes, elicit control of epithelial cell migration. TGFβ also mediates control of the events underlying stromal repair through interacting with TGFβ receptors (TGFβR) on keratocytes and fibroblasts in the stroma [8–10]. Such interaction involves control of the expression and deposition in the stromal extracellular matrix (ECM) of collagen fibers by these cell types. Furthermore, the ECM contains constituents whose constitutive expression can undergo upregulation stemming from increases in TGFβ expression during epithelial and stromal wound healing as well as embryonic tissue morphogenesis. One latter group of ECM components temporarily upregulated that affect the stromal wound healing response belong to the matricellular protein family includes tenascins, osteopontin, periostin, lumican, etc., as its members [11–19].

Tenascins are a subfamily made up of five different members including tenascin C, R, X, Y, and W. They share a unique pattern of four domains: heptad repeats, epidermal growth factor-like repeats, fibronectin type III-like repeats, and a globular domain shared with fibrinogens [20]. Amongst these, tenascin X (TNX) is of particular interest since this soluble 450 kD glycoprotein, has an important role in controlling collagen fibrillogenesis and its deposition into the ECM of some other tissues [21–23]. Mutation of the TNX gene in humans is causative of the Ehlers-Danlos syndrome. It was reported that modulation of TNX expression also modulates the effects of growth factors, i.e., TGFβ1 or vascular endothelial growth factor (VEGF) on tissue responses to external stresses [24–28].

Recent studies revealed that activation of some of the growth factor receptor linked cytoplasmic signaling cascades affect control of downstream responses through crosstalk with some of the ECM components belonging to the aforementioned matricellular superfamily. Moreover, the stromal healing responses induced by growth factor binding and stimulation of their cognate receptors are also influenced by these aforementioned ECM components.

In the present study, we determined the contribution made by TNX to corneal epithelial wound healing following tissue debridement. This characterization was undertaken because our preliminary study showed that TNX was upregulated in both the stroma and epithelium in an injured mouse cornea during healing. The results clearly showed in a TNX-deficient (KO) mouse that there was impairment of epithelial healing in association with increased infiltration of neutrophils and upregulation of reactive oxygen species (ROS) in the tissues evaluated by the accumulation of ROS-derived product (malondialdehyde) [29]. This disrupted response included a delay in wound healing, which is attributable to loss of TNX’s control of neutrophil infiltration and regulation of local ROS because systemic ablation of neutrophils or chemical scavenging ROS rescued the KO phenotype.

## Materials and methods

### Healing of an epithelial defect in in vivo mouse cornea

Each experimental protocol was approved by the DNA Recombination Experiment Committee and the Animal Care and Use Committee of Wakayama Medical University, and performed in accordance with the Association for Research in Vision and Ophthalmology Statement for the Use of Animals in Ophthalmic and Vision Research.

We first evaluated the healing of an epithelial defect in cornea in WT and KO mice. Eight-week-old C57BL/6 (wild-type, WT, *n* = 10) and KO (*n* = 10) mice of C57BL/6 background were used to analyze wound healing of mouse corneal epithelium. Under general anesthesia and topical anesthesia, a round epithelial debridement (2.0 mm in diameter) was produced in the central cornea of the right eye as previously reported [19, 30, 31]. At various healing intervals up to 36 h each cornea was photographed with green fluorescein staining and the remaining defect of the epithelium determined. The area of the fluorescein-stained epithelial defect was manually calculated from pictures. The mean value of the vertical and horizontal diameters was first obtained for determining the area by regarding the defect as a round shape. Data were analyzed by using ANOVA.

### Histology and immunohistochemistry

We then observed the cell distribution and wound healing-related components in the healing tissue by histology and immunohistochemistry. In another experimental epithelial debridement series, the mice of both genotypes were allowed to heal for 6, 12, 24 and 36 (WT mice only) h. WT (*n* = 5) and KO mice (*n* = 4) were used. The animals were sacrificed by CO_2_ asphyxia and cervical dislocation as previously reported [19, 30, 31]. Affected eyes at each timepoint were fixed in 4% paraformaldehyde in 0.1 M phosphate buffer for 48 h. Specimens were processed for hematoxylin and eosin (HE) staining and immunohistochemistry as previously reported [19, 30, 31]. The primary antibodies used are listed in Table 1. Reaction of immune-peroxidase and diaminobenzidine was performed to visualize antibody complexes.

**Table 1:** Antibodies used for immunohistochemistry and western blotting.

Antigen	Monoclonal/polyclonal	Company	Dilution in PBS
tenascin X (M23 antibody)	Rabbit polyclonal	Shimane University, Japan	1:50
IL-6	Rat monoclonal	R&D systems, USA	1:100
PCNA	Mouse monoclonal	Abcam, USA	1:100
Phospho p38	Mouse monoclonal	Santa Cruz Biotechnology, USA	1:100
MMP2	Rabbit polyclonal	Bioss Antibodies, USA	1:200
MMP9	Rabbit polyclonal	Proteintech, USA	1:100
Malondialdehyde	Rabbit polyclonal	Abcam, USA	1:1000

*IL* Interleukin, *PCNA* proliferating cell nuclear antigen, *MMP* matrix metalloproteinase.

### Western blotting

We then quantified the accumulation level of malondialdehjyde, a product related to ROS activity, in healing corneal epithelium of both genotypes of mice. Corneal epithelium of an uninjured cornea (*n* = 4 in each genotype) and healing one (*n* = 4 in each genotype) at 24 h post-wounding of both genotypes of mice were collected in sample buffer. The specimens were processed for routine western blotting as previously reported.

### Real-time reverse transcription-polymerase chain reaction (RT-PCR) in in vivo samples

Wound healing-related gene expression pattern was evaluated by real-time RT-PCR. The corneal epitheliums were harvested 6 and 12 h after debridement from either uninjured or injured corneas debrided corneas at later of either WT or KO mice. This procedure was also initially performed on 10 WT and 10 KO uninjured corneal samples collected immediately after 6 h debridement to obtain RNA samples as well as those obtained from 10 WT and 10 KO corneas at 12 h after performing this procedure. Two cornea samples were pooled into one tube. Total RNA extraction and TaqMan real-time RT-PCR were carried out to semi-quantify the expression level of targets as shown in Table 2. TaqMan primers (Applied Biosystems, Drive Foster City, CA, USA) used are listed in Table 2. Data were statistically analyzed by employing ANOVA.

**Table 2:** Primer sets for real time RT-PCR.

Target of mouse mRNA	#
IL-6	Mm01210732_gl
TGFβ1	Mm03024053_ml
MPO	Mm00447886_ml
F4/80	Mm00802529_ml
GAPDH	Mm03302249_gl

*TGFβ* Transforming Growth Factor β, *MPO* Myeloperoxidase, *GAPDH* Glyceraldehyde-3-phosphate dehydrogenase.

### Effect of systemic neutrophil depletion of on epithelial debridement healing

To examine if accelerated infiltration of neutrophils in tissue impairs healing of an epithelial defect in a KO cornea, neutrophils were systemically depleted by administration of a specific antibody. KO mice (*n* = 6 in each group) received rat anti-mouse Ly6G/Ly6C (Gr-1) antibody (50 mg/100 ml PBS, Bio X Cell, Lebanon, NH, USA) or rat anti-mouse IgG2b antibody as the control (50 mg/100 ml PBS, Bio X Cell, Lebanon, NH, USA) as previously reported [32]. After 3 days a round epithelial defect was created in a central cornea of the KO and WT mice (*n* = 6) and allowed to heal. At 18 h post-debridement the remaining defect was stained with fluorescein green and photographed. The size of the defect was statistically analyzed as described above. The mice were sacrificed and Giemsa staining examined blood samples to check neutrophil depletion. Immunohistochemistry evaluated malondialdehyde formation in eyes at 24 h post debridement.

### Epithelial healing in a KO mouse with systemic administration of N-Acetyl-L-cysteine (NAC)

NAC was used to scavenge ROS in mice. Twelve KO mice of 8-week-old with an epithelial defect was treated with either of NAC (Sigma Aldrich, St. Louis, MO, *i. p*., 200 mg/kg in 0.1 ml/10 g solution/ body weight, *n* = 6) or saline (*n* = 6). Immediately after the treatment, debridement of corneal epithelium was produced as the way above mentioned in one eye of these 12 KO mice [33–35]. Six WT mice were also processed for epithelial debridement. At 18 h post-debridement the size of the remaining epithelial defect was evaluated in photographs of fluorescein-stained corneas. Then, the animals were killed and each eye was processed for immunohistochemistry for malondialdehyde.

## Results

### TNX expression pattern in cornea

**Fig. 1:**
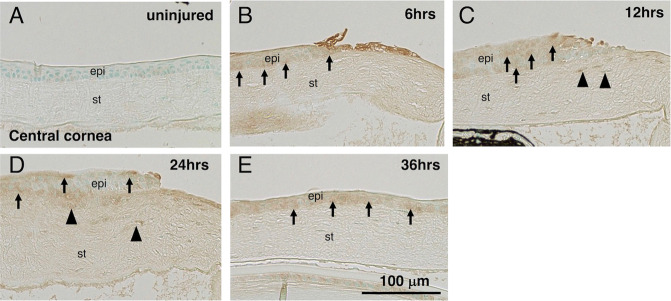
Immunohistochemical detection of expression pattern of tenascin X protein in a mouse cornea. **A** Tenascin X was not detected in the epithelium and stroma of central cornea. Frames (**B**–**E**) shows immunohistochemical detection of tenascin X at 6, 12, 24 and 36 h, respectively. Healing epithelium upregulated expression of tenascin X protein up to 36 h post-debridement (arrows). Cells in the stroma (arrowheads) transiently upregulated tenascin X with the peak at 24 h post-debridement (**D**), and then declined the expression at 36 h (**E**). Bar, 100 μm; epi epithelium, st stroma.

Immunostaining detected TNX expression in the peripheral stroma, but not in the central, cornea of an uninjured animal as previously reported (data not shown) [36]. However, during the epithelial healing process TNX protein expression was evident up to 36 h (Fig. 1). Stromal cells transiently upregulated TNX and they reached a peak at 24 h post-debridement (Fig. 1D).

### Healing of an epithelial debridement

**Fig. 2:**
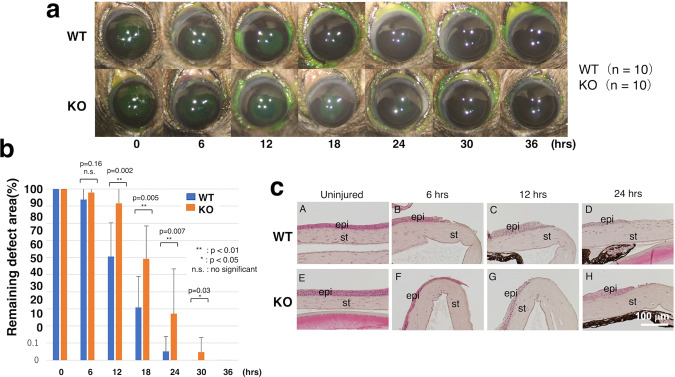
Healing of an epithelial debridement in a mouse cornea. **a** Green fluorescein staining detects an epithelial defect in each cornea. A round defect of 2.0 mm in diameter in corneal epithelium gradually became smaller and resurfaced around at 24 h in WT mice. The area of the remaining epithelial defect was indicated by the white dotted lines in Supplememtary Fig. 1. Reepithelialization was significantly delated in a KO mouse from at 12 h until 30 h post-epithelium debridement. **b** Statistical analysis detected statistical difference in the % remaining defect between two genotypes of mice at 12, 18, 24 and 30 h post-debridement. **c** Hematoxylin-eosin staining histology showed no obvious difference of keratocyte distribution in the stroma in both wild-type (WT) and KO mice (**A**, **E**). The stroma beneath the defected epithelium lacked keratocytes at 6 h in WT and KO mice (**B, F**). The cellular components re-appeared in the central stroma of both genotypes of mice at 12 (**C, G**) and 24 (**D**, **H**) h. Histology overall did not indicate obvious difference of cellular distribution between WT and KO mice. Bar, 100 μm; epi epithelium, st stroma.

A round corneal epithelial defect gradually waned after debridement and completely resurfaced itself at around 24 h in the WT mice. Reepithelialization was significantly delayed in a KO mouse from 12 h and up until 30 h. (Fig. 2a, b, Supplementary Fig. 1).

Corneal structure was unchanged by the loss of TNX expression (Fig. 2c). HE staining histology showed that the stroma situated beneath the denuded epithelial area lacked keratocytes at 6 h in both WT and KO mice (Fig. 2c). Nevertheless, they reappeared in the central stroma at 12 and 24 h. However, HE staining did not indicate any obvious difference in cell distribution between the WT and KO mice (Fig. 2c).

### Inflammatory cell infiltration in cornea

**Fig. 3:**
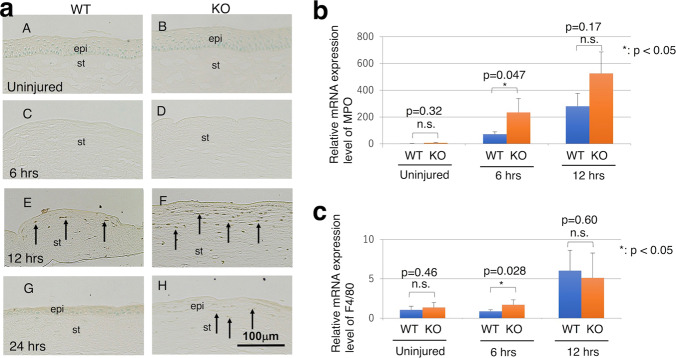
Inflammatory cell infiltration in cornea of a tenascin-null (KO) mouse. **a** Immunohistochemistry by using anti-myeloperoxidase (MPO) antibody does not detect neutrophils in both WT and KO corneas under the uninjured condition and at 6 h post-epithelial debridement. Then, at 12 h, neutrophils were distributed in the denuded stroma of both genotypes of mice. The number of the neutrophils seems more in the KO stroma as compared with WT stroma. At 24 h post-debridement neutrophils were not observed in the WT stroma, while a few cells were seen in the KO stroma. Bar, 100 μm; epi epithelium, st stroma. **b** Real-time RT-PCR indicated that infiltration of myeloperoxidase (MPO)-positive neutrophils was significantly accelerated by the loss of tenascin X at 6 h. Statistical significancy in the difference of the level of myeloperoxidase (MPO) expression was not detected at 12 h post-debridement. **c** Real-time RT-PCR indicated that Macrophage invasion was significantly accelerated by the loss of tenascin X at 6 h. Statistical significancy in the difference of the level of F4/80 expression was not detected at 12 h post-debridement.

Immunohistochemistry suggested that infiltration of neutrophils might be more marked in the KO corneal stroma as compared with a WT stroma at 18 h post-debridement (Fig. 3a). It failed to show up the difference of the distribution of macrophages in the stroma when compared with a healthy untreated tissue during healing interval (data not shown). Thus, we then performed real-time RT-PCR to further semi-quantitate infiltration of neutrophils in tissue by using real time-RT-PCR.

Real-time RT-PCR indicated that the loss of TNX at 6 h significantly accelerated MPO-positive neutrophil infiltration. However, there was no significant difference in MPO expression levels at 12 h between the KO and WT genotypes (Fig. 3b). Macrophage infiltration into the healing epithelial cells was the same in the WT and KO mice (Fig. 3c).

### Malondialdehyde accumulation in the epithelium-debrided cornea

**Fig. 4:**
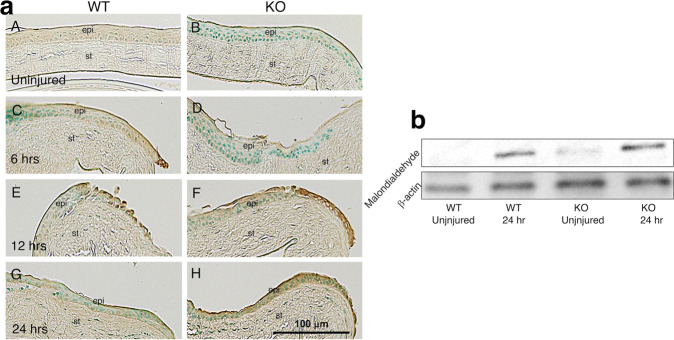
Accumulation of malondialdehyde in the epithelium-debrided cornea. **a** Uninjured epithelium of wild-type (WT, **A**) and tenascin X-null (KO, **B**) mice was faintly labeled with an antibody against malondialdehyde, a lipid peroxidated product. The spreading edge of the WT healing epithelium was positive for malondialdehyde at 6 (**C**) and 12 (**E**) hrs and staining intensity decreased in the epithelium at 24 h (**G**). In a KO mouse malondialdehyde staining in the epithelium was similar to that in a WT mouse at 6 h (**D**). Then, staining intensity was more marked in KO epithelia as compared with WT ones at 12 (**F**) and 24 (**H**) h. Bar, 100 μm; epi epithelium, st stroma. **b** Western blotting. The protein levels of malondialdehyde in an uninjyured corneal epithelium and the protein levels at 24 h after injury in KO mice were higher than those in WT mice.

Malondialdehyde immunostaining was faint in the uninjured epithelium of the WT and KO mice. At the spreading edge of epithelial cells moving inward to close a defect, there was malondialdehyde immunostaining at 6 and 12 h whose staining intensity decreased and at 24 h. In KO mice, malondialdehyde staining was similar to that in its WT counterpart. Subsequently, at later times its staining intensity was more marked in KO epithelia than in the WT mice at 12 and 24 h (Fig. 4a).

Western blotting showed that the protein level of malondialdehyde in an uninjured corneal epithelium and that at 24 h post-injury of KO mice was higher as compared with those of WT mice (Fig. 4b).

### Neutrophil ablation rescued the impairment of epithelial healing in a KO cornea

**Fig. 5:**
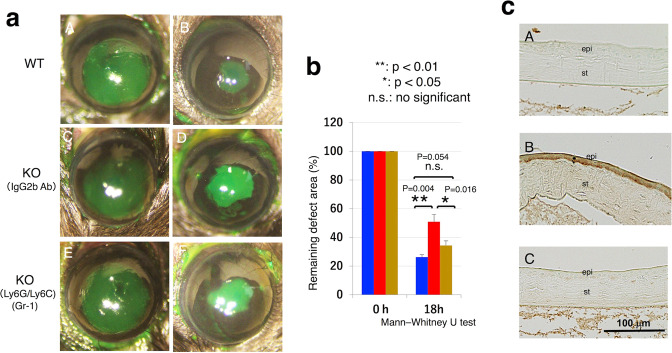
Neutrophil ablation rescued the impairment of epithelial healing in a KO cornea. **a** We examined if systemic ablation of neutrophil affects wound healing of an epithelial debridement 2.0 mm in diameter (**A**, **C**, **E**) in a tenascin X-null (KO) mouse cornea. At 18 h post-epithelial debridement, the % size of the remaining defect was smaller in a mouse treated with anti-mouse Ly6G/Ly6C (Gr-1) antibody (**F**) as compared with that in a mouse treated with a control antibody (**D**). There was no significant difference in the size of the 18 h-defect between the test group of animals (**F**) and a WT mouse (**B**). Bar, 100 μm. **b** Graph indicates % size of the remaining defect in each condition of mice. **c** Immunohistochemistry in corneas of each eyeball enucleated at 24 h showed that malondialdehyde was faintly detected in regenerated epithelium of a WT mouse (**A**). Marked immunoreactivity for malondialdehyde was detected in the epithelium of a healing KO mouse (**B**), while it’s the staining was much less marked in the epithelium of a healing neutropenic KO mouse treated with anti-mouse Ly6G/Ly6C (Gr-1) antibody (**C**). Bar, 100 μm; epi epithelium, st stroma.

Giemsa staining of blood smears failed to detect neutrophils in Gr-1 antibody-treated mice (Supplementary Fig. 2). At 18 h post-epithelial debridement, the size of the remaining defect was significantly smaller in a mouse treated with anti-mouse Ly6G/Ly6C (Gr-1) antibody as compared with a mouse injected instead with a control antibody (Fig. 5a, b). The sizes of the 18 h-defect were not different from one another between a WT neutrophil depleted and a WT control mouse (Fig. 5a, b). The same type of analysis was performed on corneas of each eyeball enucleated at 24 h. Malondialdehyde immunostaining was faint in the epithelial cells following healing in a WT mouse. More marked malondialdehyde immunoreactivity was detected in the epithelium of a healing KO mouse, while its staining was much less prominent in the healing epithelium of a neutropenic KO mouse (Fig. 5c).

### Reversal of the impairment of epithelial healing in a KO mouse by systemic administration of NAC

**Fig. 6:**
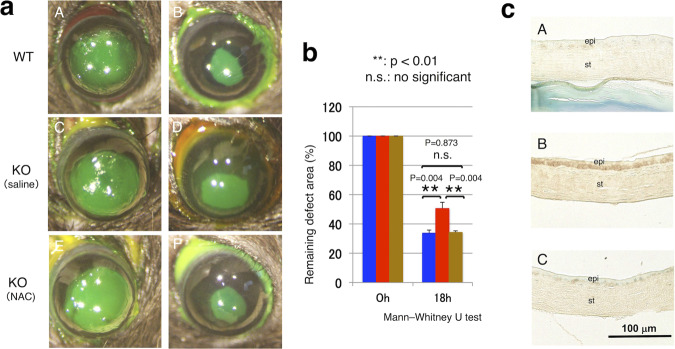
Scavenging reactive oxygen species (ROS) by systemic administration of N-Acetyl-L-cysteine (NAC) rescued the impairment of epithelial healing in a KO cornea. **a** We examined if scavenging ROS by systemic administration of NAC affects wound healing of an epithelial debridement 2.0 mm in diameter (**A**, **C**, **E**) in a tenascin X-null (KO) mouse cornea. At 18 h post-epithelial debridement, the size of the remaining defect was smaller in a mouse treated with systemic NAC administration (**F**) as compared with that in a mouse treated with saline (**D**). There was no significant difference in the size of the 18 h-defect between NAC group of animals (**F**) and a WT mouse (**B**). Bar, 100 μm. **b** Graph indicates % size of the remaining defect in each condition of mice. **c** Immunohistochemistry in corneas of each eyeball enucleated at 24 h showed that malondialdehyde was faintly detected in regenerated epithelium of a WT mouse (**A**). Marked immunoreactivity for malondialdehyde was detected in the epithelium of a healing KO mouse (**B**), while it’s the staining was much less marked in the epithelium of a healing neutropenic KO mouse treated with NAC (**C**). Bar, 100 μm; epi epithelium, st stroma.

Systemic application of NAC reversed the impairment of healing of an epithelial defect in a KO mouse. At 18 h post-debridement the % remaining defect in a KO mouse treated with systemic NAC was significantly smaller as compared with a KO mouse with saline (Fig. 6a, b). There was no statistical difference of the size of the remaining epithelial defect between a WT mouse and a NAC-treated KO mouse at this timepoint (Fig. 6a, b).

Immunohistochemistry in samples at 24 h showed that the loss of TNX markedly upregulated malondialdehyde expression in healing corneal epithelium as compared with a WT mouse. Such increase of the malondialdehyde was canceled by NAC treatment in a KO mouse (Fig. 6c).

## Discussion

In the current study we show that TNX upregulation is associated with epithelial renewal since loss of its function dramatically impaired injured corneal epithelium wound healing induced by epithelial debridement in mice. The loss of TNX attenuated wound healing of corneal epithelium through inducing increases in neutrophil infiltration from the periphery since both antibody-induced neutropenia rescued the impairment of wound closure of a KO cornea. Increased ROS-derived product in a KO healing epithelium strongly suggested that over-activation of ROS suppressed epithelial repair. Indeed, scavenging ROS rescued the impairment of epithelial healing in a KO mouse. Taken together, we concluded that neutrophil-derived ROS attenuated epithelial healing in the absence of TNX. Tenascin C is another tenascin family member that is also reportedly upregulated during wound healing, but it is not actively involved in reversing losses in epithelial integrity [37].

Upregulation of wound healing-related growth factors/cytokines is critical to inducing corneal epithelial renewal during wound healing [38]. However, loss of TNX function did not affect any differences in mRNA expression levels of and immunohistochemical protein expression level of TGFβ1 and IL-6 in both genotypes of mice (Supplementary Fig. 3), indicating that neither IL-6 nor TGFβ1 contribute to the decline in epithelial cell migration in the absence of TNX. Growth promoting receptor activation mediates increases in cell proliferation and migration during wound healing through stimulating different branches of the mitogen activated protein kinase (MAPK) cascade. In the current study we did not see difference of cell proliferation activity between WT and KO healing epithelia (Supplementary Fig. 4). We previously reported that TGFβ-driven p38 activation is critical to epithelial cell migration in a mouse cornea. In the present study the loss of TNX did not affect the activation level of p38 in the healing corneal epithelium post-debridement (Supplementary Fig. 4).

MMPs reportedly modulate epithelial healing [39]. Loss of MMP9 accelerated healing of an epithelial defect in a mouse cornea [40]. We previously documented that loss of TNX expression accompanies MMP2 and MMP9 upregulation in melanoma tumor cells [27]. Furthermore, we also showed that loss of TNX function accelerated MMP2 upregulation through increases in the activity of both the c-Jun N-terminal kinase and protein tyrosine kinase phosphorylation pathways [41]. However, in a mouse cornea, our western blotting showed no obvious difference of expression level of both MMP2 and MMP9 in healing corneas between WT and KO mice (Supplementary Fig. 5). The difference of the expression level of MMPs might depend on cell linage.

Since inflammation is a correlate of wounding, we performed basic HE histological and immunohistochemistry analysis to evaluate if inflammatory cell infiltration was identifiable in the stroma beneath the epithelial defect. Immunohistochemical observations and real-time RT-PCR that the loss of TNX enhanced neutrophil infiltration in the healing cornea post- epithelial debridement. Immunohistochemistry showed that stroma of an uninjured WT cornea does not contain TNX, while during post-debridement healing, migrating epithelial cells, stromal cells and matrix stained for TNX. Neutrophil migration or infiltration is reportedly modulated by changes in ECM components. For example, lumican, one of the core proteins of corneal keratin sulfate proteoglycan, is required for inducing adequate increases in neutrophil infiltration to counter external stress induced by bacterial infection or tissue injury [42]. The underlying mechanism accounting for post-debridement increases in neutrophil infiltration in the KO stroma requires future clarification. Our unpublished data showed that the thinner subepithelial basement membrane in a KO cornea as compared with that in a WT one. However, it is to be uncovered if such alteration of the basement membrane affects cell migration. It is noteworthy that gene knockout of tenascin C, another tenascin family member, accelerates neutrophil population in an injured tissue [43, 44]. Loss of TNX function also accelerates migration of non-hematopoietic linage cell types such as neoplastic epithelial cells [27]. It is possible that declines in ECM integrity and collagen content in KO mice increase stromal porosity. Such a change may increase neutrophil permeance through the underlying the stroma [45].

Injury subsequently reduces the antioxidant status as a consequence of an upregulation of ROS generation. ROSs are critical regulators of each step of tissue repair: while low concentrations of ROS generation are cell survival signaling, excessive production of ROS causes oxidative damage and impairs tissue repair, which is one of the main causes of refractory chronic wounds [29, 46–52]. Malondialdehyde is a lipid peroxidation-derived product whose expression level is frequently used as a readout to evaluate the level of oxidative stress. Accordingly, increases in its expression level reportedly in tissue is reportedly negatively correlates with tissue repair progression in skin or cornea [29, 46–49, 51, 53]. Immunohistochemistry and western blotting showed that the loss of TNX upregulated malondialdehyde accumulation in corneal epithelium. This finding indicates that the loss of TNX upregulated ROS generation and that oxidative stress activity in the local tissue was more marked in a KO mouse as compared with a WT mouse. Enhanced neutrophil leukocyte infiltration in a KO tissue could be a source of excess ROS because it is one of the major origins of ROS in an injured or infected tissue [54, 55].

Roles of ROS in epithelial tissue repair reportedly depend on its level. Adequate level of ROS is considered to be beneficial to cell proliferation acceleration [46]. On the other hand, excessive ROS could impair the epithelial tissue repair via inhibition of cell migration and proliferation [29]. In vivo behaviors of epithelial cells under tissue repair process are modulated by multiple factors. Cell migration and proliferation are both affected the size of the defect to be recovered, and thus, we comprehensively evaluated the in vivo tissue repair activity of corneal epithelium in the absence of TNX in the present study. We hypothesized that ROS over-activation and an impairment of epithelial healing could be attributable to an excess infiltration of neutrophils in a KO cornea tissue, although it is controversial whether infiltration of neutrophils into an injured local tissue accelerates or delays wound healing [56–61]. To establish this conclusion, we systemically depleted circulating neutrophils by injecting anti-Gr-1 antibody which is a neutrophil-specific antibody. This antibody reversed declines in epithelial wound healing in a KO cornea, which was associated with a decline in oxidative stress based on less malondialdehyde staining in epithelial cells participating in the healing response to injury. Therefore, increases in neutrophil infiltration during wound healing in KO and the WT mice contribute to retarding responses promoting this response to injury.

In another study, neutrophil depletion with the same aforementioned antibody instead failed to reverse the decline in corneal epithelial regeneration induced by epithelial debridement in hemeoxygenase-2 (HO-2)-null mice [53]. Even though neutrophil infiltration into the wounds was greater in the WT than in the HO-2-null mice, neutrophil depletion inhibited corneal wound closure by 60% and 85%, respectively irrespective of differences in the genotype. This failure of neutrophil depletion to reverse declines in wound healing in the HO-2-null mice suggests that loss of HO-2 gene ablation function may have unique effects on cellular events controlling the cell redox status, which may be absent in the TNX-deficient mice. There is substantive evidence showing that the phenotypes during epithelial healing of HO-2-null and TNX-deficient mice are markedly different from one another. Loss of HO-2 function impairs wound healing and exacerbates inflammation following injury. These effects stem from the inability of cells to organize a basal tone of anti-inflammatory signals that are a prerequisite for establishing an acute rather than a chronic non self-limiting inflammatory response to injury-induced oxidative stress that disrupts wound healing. In other words, subsequent to a loss of HO-2, a key element in the hemoxygenase system, a needed anti-inflammatory circuit cannot be activated to terminate an inflammatory response to injury. Instead the response persists and becomes chronic driven by a number of different types of activated immune cells besides neutrophils [62, 63]. In contrast, in the mice that lacks TNX, there are no indications that such a loss in function alters mediators controlling termination of an inflammatory response to injury. Instead the effects of loss of TNX function are limited to alterations in the structural framework of the ECM. The collagen fibers elaborated by fibroblasts are small and sparse and the ECM is unevenly packed around myelin sheaths. Such effects are associated with reduced tensile strength and collagen fiber deposition in the skin. These changes are limited to impairing the integrity of the collagen matrix [45]. This difference between the effects of loss of TNX and HO-2 function are consistent with the fact that following neutrophil depletion only the debrided HO-2-null corneas became chronically inflamed and manifested amplified long-lasting increases in the production of proinflammatory chemokines that are also associated with persistent neovascularization and decreased tissue repair.

In the current study we finally examined if ROS presumably derived from neutrophils certainly impaired epithelial healing by using a strategy to scavenge ROS in mice with an epithelial defect in cornea. The results showed that systemic administration of NAC reversed the impairment of epithelial healing by the loss of TNX in association with reduction of expression level of malondialdehyde in healing epithelium. This finding clearly indicates that delayed healing in corneal epithelium in a KO mouse is attributable to the over-activation of ROS.

In conclusion, our results indicate that the mechanism of perturbed epithelial healing in a KO mouse includes accelerated population of neutrophils and ROS over-activation in the epithelium-debrided cornea.

## Supplementary information

Supplementaly files
