# Supplementary material for: Impairment of corneal epithelial wound healing is association with increased neutrophil infiltration and reactive oxygen species activation in tenascin X-deficient mice
Source: Lab Invest. 2021 Mar 29;101(6):690–700. doi: 10.1038/s41374-021-00576-8 (PMC8137452; doi:10.1038/s41374-021-00576-8)
Supplement: Supplementary file 1 — Supplementaly files [file 41374_2021_576_MOESM1_ESM.pdf]

Supplemental data

Supplemental figure 1

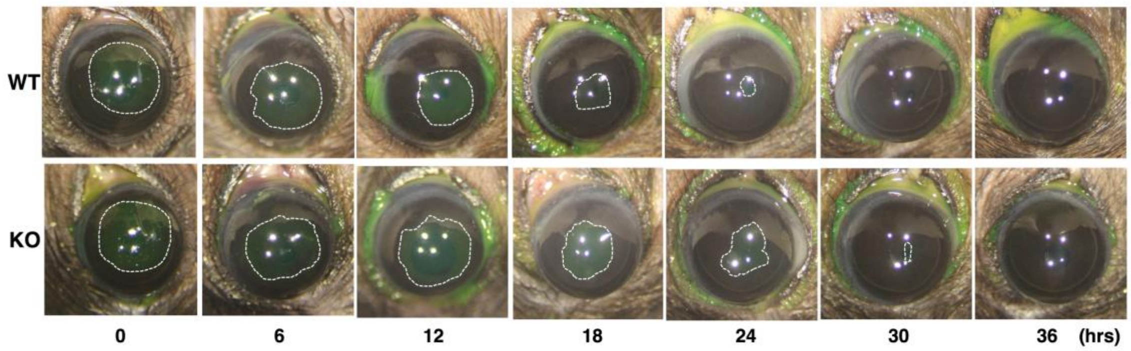

Supplemental figure 1.

The area of the remaining epithelial defect shown in the frame a of Figure 1 was indicated by the white dotted lines.

Supplemental figure 2

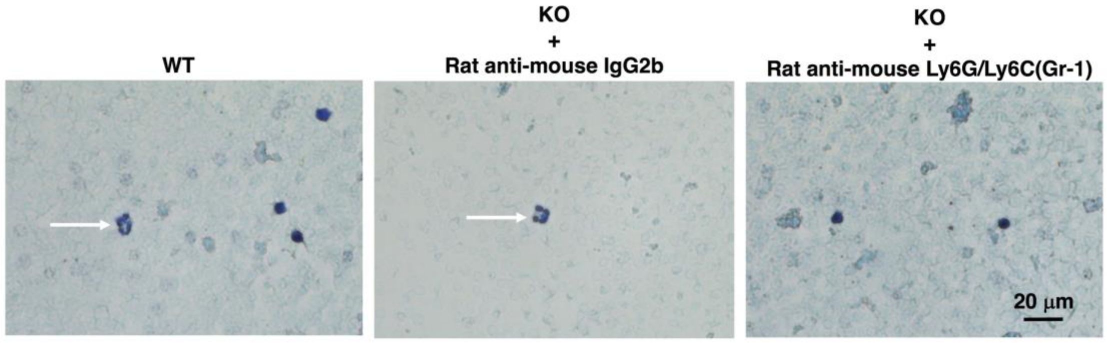

Supplemental figure 2.

Neutrophils were systemically depleted by administration of a specific antibody. KO mice (n = 6 in each group) received rat anti-mouse Ly6G/Ly6C (Gr-1) antibody (50 mg/ 100 ml PBS, Bio X Cell, Lebanon, NH, USA) or rat anti-mouse IgG2b antibody as the control (50 mg/ 100 ml PBS, Bio X Cell, Lebanon, NH, USA) as previously reported [31]. After 3 days a round epithelial defect was created in a central cornea of the KO and WT mice (n = 6) and allowed to heal. At day 3 Giemsa staining of blood smears failed to detect neutrophils in Gri-1 antibody-treated mice. Bar, 20 μm.

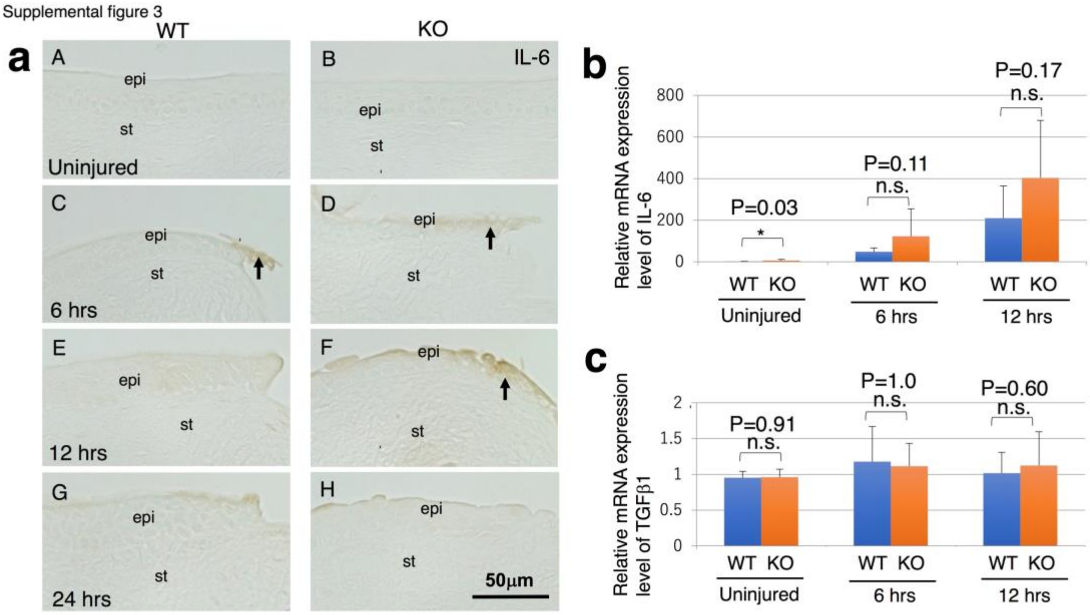

Supplemental figure 3.

Expression patterns of interleukin 6 (IL-6) and transforming growth factor β1 (TGFβ1) in epithelium-defected cornea.

The factors are reportedly involved in the healing of corneal epithelium in mice.

a. Immunohistochemistry detected upregulation of IL-6 protein in healing epithelium (arrows) of both WT (A, C, E, G) and KO (B, D, F, H) mice with no obvious difference of staining intensity through the healing interval at 6 (C, D), 12 (E, F) and 24 (G, H) hrs, while an uninjured epithelium does not stain for IL-6 in both WT (A) and KO (B) mice. Bar, 100 µm; epi, epithelium; st, stroma. **b**. Real-time RT-PCR indicated more marked expression of IL-6 in a KO uninjured tissue, but not at 6 and 12 hrs post-wounding. c. There is no difference of expression level of TGFβ1 mRNA in a healing tissue between both genotypes.

Supplemental figure 4

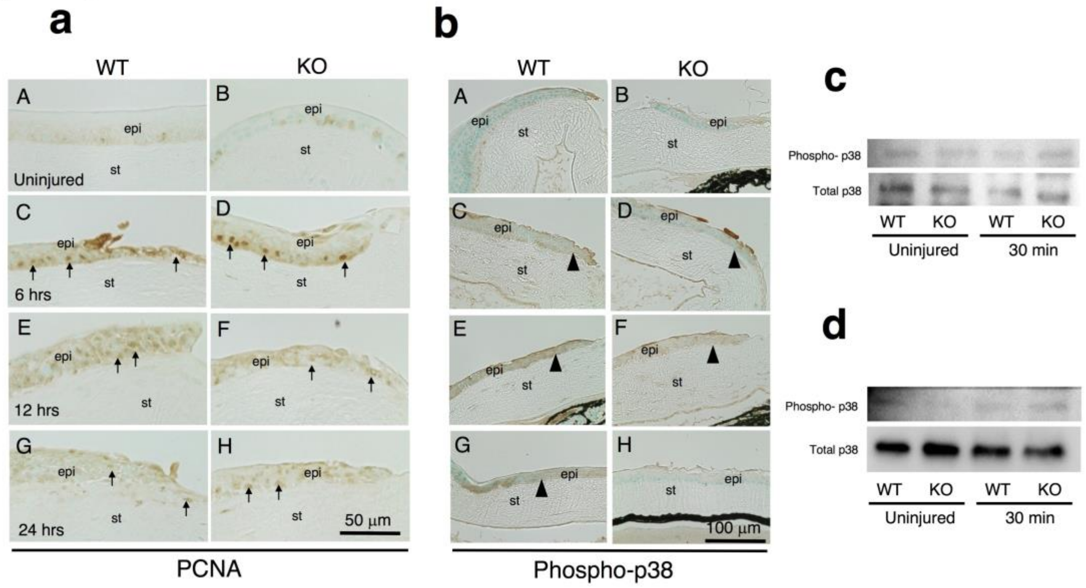

Supplemental figure 4.

Cell proliferation and migration-related signal, p38, in healing epithelium.

a. Epithelial debridement upregulated proliferation as detected by proliferating cell nuclear antigen (PCNA)-immunohistochemistry in remaining epithelium in WT (**A**, **C**, **E**, **G**) and KO (**B**, **D**, **F**, **H**) mice (arrows). PCNA-immunohistochemistry detected that epithelial cell proliferation was upregulated during wound healing. The loss of TNX expression did not seem to suppress epithelial cell proliferation. Bar, 50 µm; epi, epithelium; st, stroma. **b**. We examined if lacking TNX affects the p38 phosphorylation status whose modulation is an essential encoder of receptor control of epithelial cell migration. We previously reported that phospho-p38 formation is dependent on TNX expression which in turn is required for cell adhesion in epithelial cell culture [63]. Immunohistochemistry shows that healing epithelium (arrowheads) upregulated phospho-p38 during healing interval at 6, 12 and 24 hrs post-debridement as compared with an uninjured epithelium of both genotypes of mice. Immunohistochemical intensity for phospho-p38 was similar between WT (**A**, **C**, **E**, **G**) and KO (**B**, **D**, **F**, **H**) mice. Bar, 100 µm; epi, epithelium; st, stroma. **c, d**. Western blotting further showed no obvious difference of phosphorylation level of p38 between two genotypes of mice at 30 min (**c**) and 12 hrs (**d**) post-epithelial debridement. Taken together, the current in vivo analysis showed that during epithelial healing phospho-p38 underwent upregulation to similar levels in both the TNX KO and the WT mice.

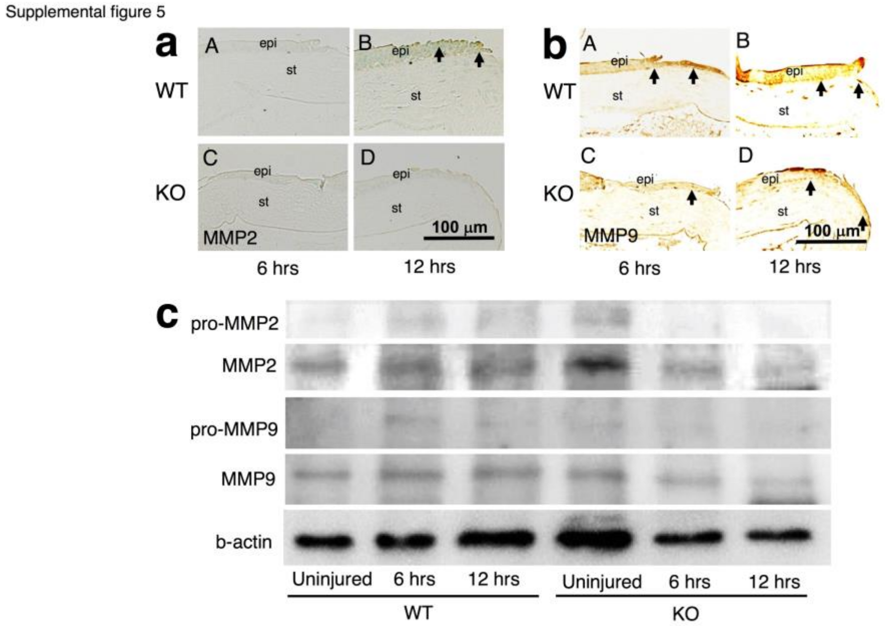

Supplemental figure 5.

Expression of matrix metalloproteinase (MMP) 2 and 9 in healing corneal epithelium.

MMPs expression levels affect control of healing rates of corneal epithelial closure during wound healing [39, 40, 41]. Healing of corneal epithelium (arrows) defect depends on the expression of MMPs. We examined the protein expression level of MPP2 and MMP9 by using immunohistochemistry and western blotting. Immunohistochemistry detected both MMP2 (**a**) and MMP9 (**b**) in WT (**A**, **B**) and KO (**C**, **D**) healing epithelia (arrows) at 6 and 12 hrs post-defect. Bar, 100 μm; epi, epithelium; st, stroma. **c**. Western blotting showed that expression of MMP2 and MMP9 was similar between WT and KO tissue at 6 and 12 hrs post-debridement.
